# A Novel Splice Site Mutation in the *FBN2* Gene in a Chinese Family with Congenital Contractural Arachnodactyly

**DOI:** 10.1007/s10528-023-10550-2

**Published:** 2023-11-14

**Authors:** Cuiping Zhang, Fengchang Qiao, Qing Cheng, Chunyu Luo, Qinxin Zhang, Ping Hu, Zhengfeng Xu

**Affiliations:** Department of Prenatal Diagnosis, Nanjing Maternity and Child Health Care Hospital, Women’s Hospital of Nanjing Medical University, Nanjing, 210004 China

**Keywords:** Congenital contractural arachnodactyly, *FBN2*, Whole exome sequencing, Splice site, Exon skipping

## Abstract

Congenital contractural arachnodactyly (CCA) is a rare connective tissue disorder characterized by arachnodactyly, multiple joint contractures, progressive kyphoscoliosis, pectus deformity and abnormal crumpled ears. *FBN2* is the only gene currently known to be associated with CCA. In this study, we report on a prenatal case presented with skeletal, cardiac and spinal malformations. And his father had elongated limbs, contractures of the proximal interphalangeal joints, high myopia and scoliosis. We conducted whole exome sequencing (WES) on the fetus-parental trio and a heterozygous variant (hg19 chr5:127,673,685, c.3598 + 4A > G, NM_001999.4) in intron 27 of the *FBN2* gene was successfully identified, inherited from the father. Reverse transcriptase-polymerase chain reaction (RT-PCR) was performed to evaluate the potential splicing effect of this variant, which confirmed that the variant caused a deletion of exon 27 (126 bp) by disrupting the splice-donor site and destroyed the 17th calcium-binding epidermal growth factor-like (cbEGF) domain. Our research not only finds the etiology of the disease in affected individuals and expands the mutation spectrum of *FBN2* gene, but also provides genetic counseling and fertility guidance for this family.

## Introduction

Congenital contractural arachnodactyly (CCA, OMIM #121,050), also known as Beals–Hecht syndrome (BHS), was first described by Rodney Beals and Frederick Hechet in 1971 (Jurko et al. 2013). CCA is a rare connective tissue disease inherited in an autosomal dominant manner with variable expressivity. The characteristic phenotypic spectrum of CCA includes arachnodactyly, progressive kyphoscoliosis, multiple joint contractures (elbows, knees, hips, ankles and fingers), abnormal crumpled ears and pectus deformity (Li et al. 2020). Scoliosis is the most serious complication of CCA, occurring in approximately half of patients, and is progressively worse, requiring regular follow-up and even surgical treatment (Mehar et al. 2014). Abnormal crumpled ear is the most typical phenotype and appear as flattened helices (Courtens et al. 1998). Significantly, the performance of the joints and ears will be gradually improved over time, whereas scoliosis and long-bone overgrowth may become more prominent (Callewaert 1993; Guo et al. 2016). Clinically, CCA can be divided into two types: classic CCA and severe/lethal CCA. In addition to the above typical symptoms described, severe/lethal CCA presents as cardiovascular and/or gastrointestinal anomalies that is a rare infant phenotype, comprising atrial or ventricular septal defects, interrupted aortic arch and intestinal malrotation (Callewaert 1993; Babcock et al. 1998).

CCA is caused by mutations in the *FBN2* gene located on chromosome 5q23.3. So far, *FBN2* is the only gene known to be associated with CCA (Hu, Li et al. 2021). *FBN2* encodes an elastin-associated microfibrillar protein named fibrillin-2 that consists of five modules: EGF-like domain, calcium-binding EGF-like (cbEGF) domain, glycine-rich domain, hybrid domain, and 8-cysteine repeat region (Hu et al. 2021). Fibrillin-2 protein are structural components of extracellular calcium-binding microfibrils in elastic fiber (Zhou et al. 2018). It has an essential function in raising the strength and elasticity of the connective tissue that sustains the body's organs and joints (Li et al. 2020). Mutations in *FBN2* gene would disrupt the stability of the protein, cause abnormal expression of fibrillin-2, eventually disrupt the normal structure of connective tissue and lead to the occurrence of CCA (Hu et al. 2021).

In our study, in order to explore the genetic etiology of skeletal abnormalities and ventricular septal defects in the fetus, we performed trio whole exome sequencing (WES) and identified a novel mutation (c.3598 + 4A > G) in the *FBN2* gene. Interestingly, through further experiments, we verified that this variant affected splicing and resulted in an in-frame deletion of exon 27, and ultimately led to the clinical phenotype of CCA.

## Materials and Methods

### Study Subjects

**Fig. 1:**
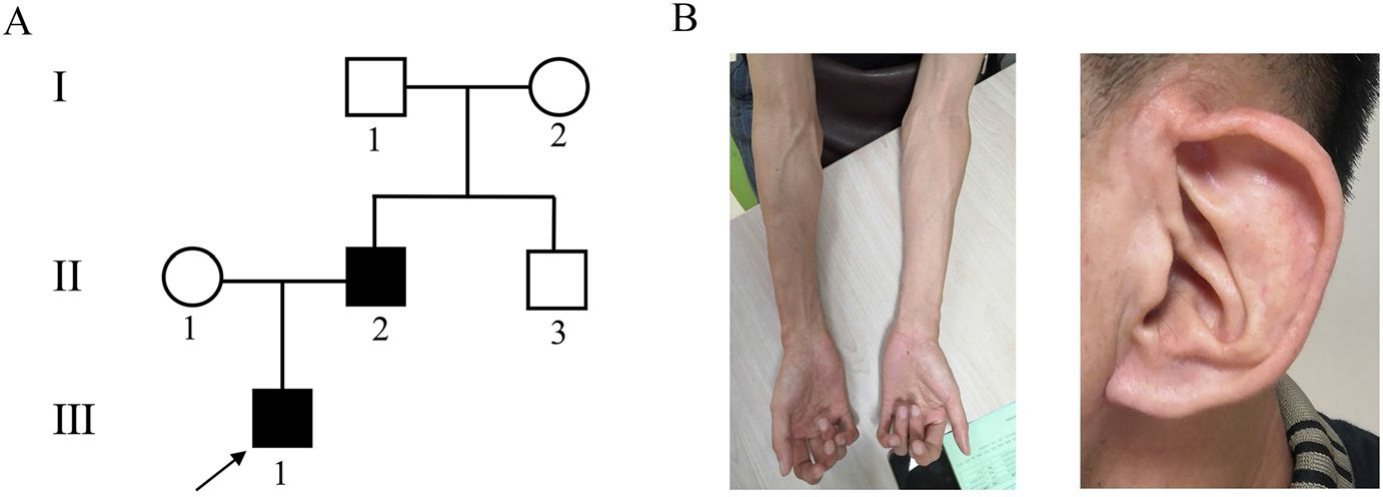
Pedigree of the congenital contractural arachnodactyly (CCA) family and imaging features of the fetal father. (**A**) Pedigree of the family with segregation of the identified *FBN2* mutation. The square represents the male, and circle represents the female. A filled symbol indicates the affected individuals and the arrow indicates the proband. (**B**) The picture illustrates contractures of the proximal interphalangeal joints, the slender arms and abnormal crumpled ears showing a "tram track" appearance in the fetal father

A 33-year-old pregnant woman was referred to our hospital for genetic counseling at 23^+5^ weeks’ gestation. The woman and her husband were non-consanguineous, with no history of infection and/or exposure to teratogens (Fig. 1A). The fetus presented with right humerus and bilateral ulnar radius curvature, continuous clenched hands, ventricular septal defect, and head circumference was about 1–2 weeks smaller than the corresponding gestational age. The sacrum is slightly wider on the longitudinal section of the spine. The father has crumpled appearance of ear helix, long slim limbs, contractures of the proximal interphalangeal joints, high myopia and scoliosis (Fig. 1B). The pregnant woman did not have any phenotypic abnormalities. Amniocentesis was performed at 24 weeks of gestation after genetics counseling. The results of chromosomal microarray analyses (CMA) were negative. Considering the abnormalities of fetal ultrasound, the couple opted to terminate the pregnancy at 27^+2^ weeks. The terminated fetus presented with elongated limbs and finger contracture. However, the couple did not agree to an autopsy. To further identify the genetic etiology, we conducted WES on the fetus and his parents.

### Whole Exome Sequencing

Genomic DNA was extracted from the fetal skin and parental peripheral blood samples using an Automated Nucleic Acid Extractor (RBC Bioscience) following standard procedures for genetic analysis. Fragment libraries were created by sheared and amplified DNA with a read length of 150 bp. After captured with IDT the xGen Exome Research Panel v1.0 and sequenced on the Illumina NovaSeq 6000 (Illumina, Inc.) platform according to the manufacturer’s protocols, the resultant reads were mapped against reference genome sequence (GRCh37/hg19) assembly using the Burrows-Wheeler Aligner (v 0.7) software package. Then remove PCR duplication with samtools (v 0.1.18). Single-nucleotide variants (SNVs) and short insertions and deletions (indels) were filtered using the Genome Analysis Toolkit 3.4 (GATK). Variants were filtered against the 1000 Genomes database (1000 genomes release phase 3, http://www.1000genomes.org/), dbSNP database (http://www.ncbi.nlm.nih. gov/projects/SNP/snp_summary.cgi) and the genome aggregation database (gnomad.broadinstitute.org) with minor allele frequency > 0.5%. Further bioinformatics analyses were performed using the SIFT (http://sift.jcvi.org/), Mutation Taster (http: //www.mutationtaster.org), Proven(http://provean.jcvi.org/index.php) and PolyPhen-2 (http://genetics.bwh.harvard.edu/pph2/). Amino acid conservation was evaluated by GERP (http://mendel.stanford.edu/sidowlab/downloads/gerp/index.html) and PhyloP (http://compgen.bscb.cornell.edu/phast/). The potential splicing effects were evaluated by using splice‐site prediction software, including Human Splicing Finder (http://www.umd.be/HSF/), MaxEntScan (http://genes.mit.edu/burgelab/maxent/Xmaxentscan_Scoreseq.html) and NNSplice (http://www.fruitfly.org/seq_tools/splice.html). Candidated variants were reanalyzed visually by Integrative Genomics Viewer (IGV) and validated by Sanger sequencing.

### RNA Transcript Analysis by RT–PCR

Total RNA was extracted from fetal tissue sample and parental white blood cells using TRIzol™ Reagent (Invitrogen; Thermo Fisher Scientific) according to the standard procedures. First-strand cDNA synthesis was conducted with 1 μg of total RNA using a PrimeScript™ RT reagent Kit with gDNA Eraser (TAKARA). To detect whether aberrant splicing patterns are occurring, we amplified PCR products using specific primers (forward primer: 5′-AATGCCGTTGCAATAGTGGC-3′ and reverse primer: 5′-CATCATAGCAGAGGCAGCGA-3′). PCR products were ultimately separated on a 2% agarose gel and validated by Sanger sequencing.

## Results

**Fig. 2:**
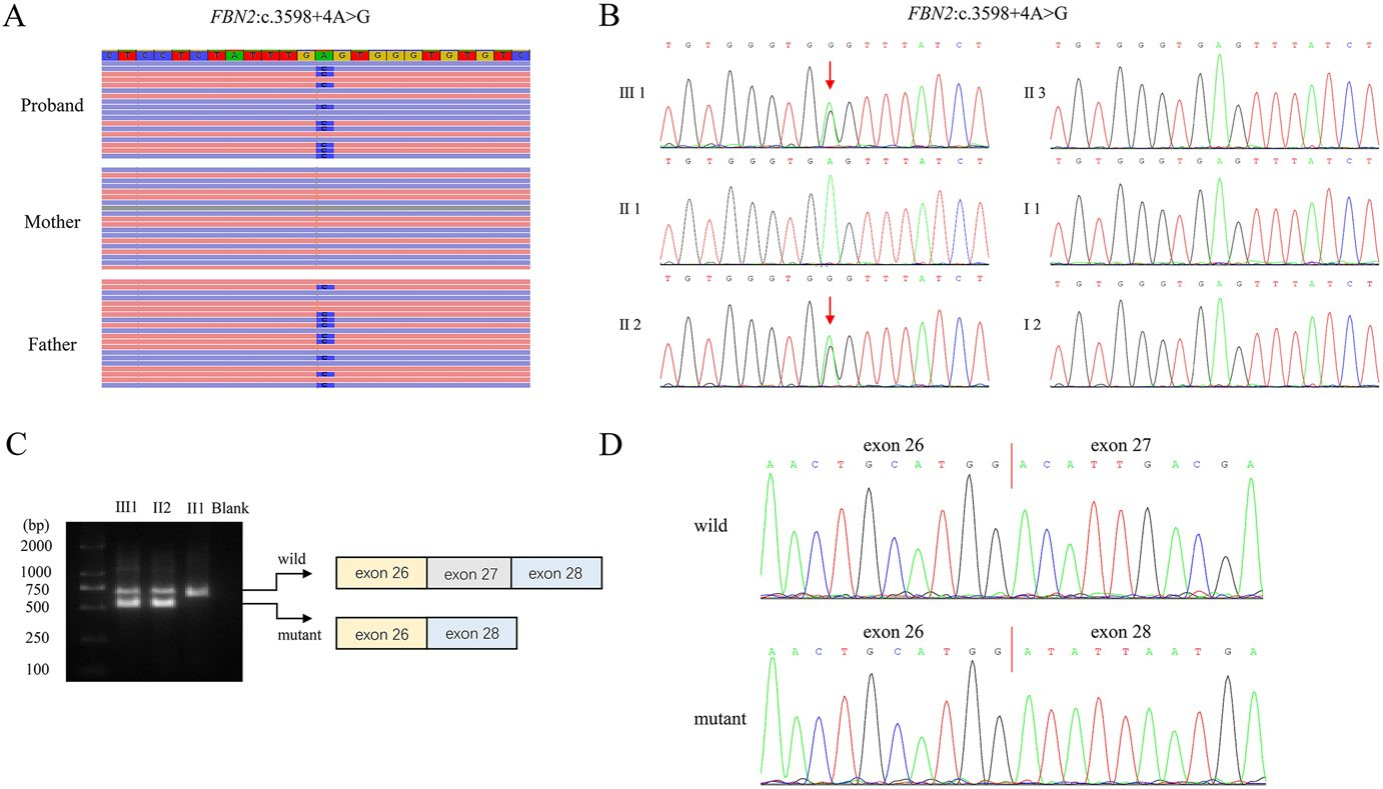
*FBN2* mutation analysis. (**A**) Visualization of the mutation in *FBN2* is shown with an integrative genomics viewer. The variant (c.3598 + 4A > G) was heterozygous in the proband and his father. (**B**) The mutation (c.3598 + 4A > G), which was only present in the fetus and his father, was validated by Sanger sequencing. (**C**) Gel image showing the PCR products of *FBN2* cDNA fragments. The wild-type and mutant PCR products were 648 bp and 522 bp fragments, respectively. (**D**) Direct cDNA sequencing of wild-type and mutant alleles in the affected individuals revealed the deletion of exon 27 (126 bp)

According to the advice of the geneticist, the fetus first underwent CMA without any pathogenic copy number variants. WES was conducted subsequently and obtained 68.79 million reads with a length of 150 bp. The average sequencing depth was 115 × and 97.91% of the whole exome target region covered at ≥ 20 × . There were 49,048 single-nucleotide polymorphisms (SNPs) in the proband, including 177 frameshift mutations, 3328 missense mutations and 957 splice site mutations. Through inheritance pattern, age of onset and in silico predictive algorithms, three phenotypic-related variants were selected. The proband carries a compound heterozygous variant with one allele containing a missense mutation of c.20131C > T (p.R6711W) and the other allele containing a missense mutation of c.6971G > A (p.S2324N) (NM_001271208.2) in the *NEB* gene. The characteristics of the disease were basically consistent with the phenotype of this case, but it was excluded due to the inconsistency with the father's symptoms. Of note, a 5′ splice site variant c.3598 + 4A > G (NM_001999.4) in the *FBN2* gene was identified both in the proband and his father, which was consistent with the clinical phenotype of the patients (Fig. 2A). The mutation was not found in ExAC, gnomAD or 1000G database (ACMG variant evidence PM2_P). In addition, we expanded the pedigree and performed variant analysis for the father’s parents, and the results confirmed that the variant was de novo (Fig. 2B) (ACMG variant evidence PS2_M). Meantime, bioinformatics predicted that this variant might affect splicing. Therefore, we performed RNA transcript analysis using specific RT-PCR to explore the implication of this aberrant variant on the splicing of *FBN2*. Agarose gel electrophoresis showed two cDNA fragments (648 bp and 522 bp) in the proband and his father, while a single amplified fragment (648 bp) in control sample (Fig. 2C). Validated by sanger sequencing, the aberrant band (522 bp) was generated by skipping of exon 27 (126 bp) (Fig. 2D) (ACMG variant evidence PS3_M). Last but not least, *FBN2* gene is compatible with the phenotype in this case, because the abnormal crumpled ears were unique to CCA patients and differentiated from other diseases (ACMG variant evidence PP4). Therefore, the identified variant c.3598 + 4A > G (NM_001999.4) in the *FBN2* gene was classified as likely pathogenic (PS3_M, PS2_M, PM2_P, PP4) according to ACMG guidelines (Roy et al. 2018).

## Discussion

In the study described here, we described a Chinese fetus with skeletal, cardiac and spinal malformations. And his father exhibited arachnodactyly, joint contractures, abnormal crumpled ears and kyphoscoliosis. Subsequently, A noncanonical splice site mutation c.3598 + 4A > G in *FBN2* was identified by WES. The symptoms of the fetus and his father were consistent with the clinical manifestations of CCA.

In patients diagnosed with CCA clinically, only 25%-75% have identifiable variants in the *FBN2* gene (Callewaert 1993). In ClinVar, we also analyzed 2276 variants associated with CCA. However, only 7.4% of variants can provide fertility guidance to patients and are recognized as pathogenic or likely pathogenic variants (113/2617). The reported types of mutations include copy number variation (CNV), missense mutations, splice site mutations, nonsense mutations and in-frame deletion.

According to reports in the literature, the *FBN2* gene has a mutational hotspot region clustered in exons 24–35, the so-called neonatal region, which encodes the calcium-binding epidermal growth factor-like (cb-EGF) domains (Xu et al. 2020). The vast majority of mutations in previous reports were concentrated in this hotspot region until a mutation located in exon 17 in a patient with classical CCA was first reported by Callewaert et al. (2009). For the *FBN2* gene, missense mutations and splice mutations were the most common variants, accounting for 50% and 46.9%, respectively. Missense mutations are thought to weaken calcium-binding and protein folding by hindering disulfide bond formation (Gupta et al. 2002; Davis and Summers 2012; Takeda et al. 2015). Splice mutations often result in exon skipping. These pathogenic variants reduced the amount of fibrillin-2 available to form microfibrils. Decreased microfibril formation reduced the elasticity of fibers, which leads to the symptoms of CCA (Davis and Summers 2012; Sengle et al. 2015).

Here, trio-WES detected a heterozygous noncanonical variant c.3598 + 4A > G (NM_001999.4) in intron 27 of the *FBN2* gene. The variant resulted in a heterozygous deletion of exon 27 as verified by RT-PCR. The deleted exon 27 is located in the 17th cb-EGF domain. As we all know, there are 43 cb-EGF domains in fibrillin-2 protein, as determined by homology to fibrillin-1. Each of cb-EGF domain is composed of six conserved cysteine residues, which form three disulfide bridges to maintain protein stability. Calcium ions binding raises-folding stability and helps to secure the two neighboring cb-EGF domains in a relative orientation, forming a typical sheet-loop-sheet motif: two antiparallel beta-sheets bridged by a Calcium ions chelation loop (Corson et al. 1993). On the one hand, the deletion of exon 27 would disrupt the 17th cb-EGF domain of *FBN2*, affect the binding of the cb-EGF domain to calcium ions, and make fibrillin-2 more susceptible to hydrolysis; on the other hand, disruption of the 17th cb-EGF domain may alter the spatial conformation and intermolecular interactions of proteins (Xu et al. 2019), and eventually lead to CCA.

There are still some reports on single exon deletions in the *FBN2* gene. Hu et al. reported that mutations altering the cb-EGF domain in fibrillin-2 affected the formation of extracellular matrix microfibers and led to severe CCA phenotype (Hu et al. 2021). For splicing mutation, Xu et al. described a family with 9 CCA patients and identified a novel splicing variant (c.3724 + 3A > C) in intron 28 of the *FBN2* gene. The variant led to an in-frame deletion of exon 28 during transcription and disrupted a cb-EGF domain in fibrillin-2, which diminishes the stability of an antiparallel beta-sheet and ultimately disrupts the folding of fibrillin-2 (Xu et al. 2020). Earlier, Babcock et al. reported a family with phenotypic characteristics of CCA and identified a *FBN2* mutation (c.3340G > C) that altered the 5’ donor splice site consensus sequence of exon 25 and caused deletion of exon 25 (Babcock et al. 1998). Taken together with above mentioned examples, exonic deletions resulting from splicing mutations in the *FBN2* gene can lead to CCA, which is consistent with our findings.

Clinically, it is easily confused with Marfan syndrome (MFS, OMIM #154,700). These two similar syndromes are heritable connective tissue disorders in an autosomal dominant manner caused by mutations in two genes: *FBN1* and *FBN2*, respectively. They have many similar clinical symptoms, such as a so-called marfanoid appearance constituted by tall, slender, asthenic appearance and skeletal features such as arachnodactyly, dolichostenomelia, pectus deformities and kyphoscoliosis, but patients with CCA do not have the ocular and cardiovascular complications that characterize MFS (Frederic et al. 2009; Zhou et al. 2018). The incidence of MFS is reported to be 1:10,000, however, the estimated incidence of CCA is unclear because of the overlap with MFS in phenotype but seems to be lower than that of MFS (Putnam et al. 1997). Therefore, genetic testing is an effective supplement for the diagnosis of CCA.

Furthermore, CCA was diagnosed prenatally in our case. Although the mutation was inherited from the father, the father had a milder phenotype while ultrasound abnormality of fetus present before delivery. Our finding not only clarify the etiology of the fetal anomaly, but also help guide the couple's next pregnancy. Eventually, they gave birth to a healthy baby through pre-implantation genetic testing (PGT).

In summary, this study identified a heterozygous noncanonical variant c.3598 + 4A > G (NM_001999.4) in intron 27 of *FBN2* gene in a fetus and his father who were both classified as classic CCA. Our results indicated that splicing error of exon 27 in *FBN2* gene and disruption of 17th cb-EGF domain was the cause of CCA, furthering the understanding of the molecular basis of this disorder. Thus, the variant identified in our case not only finds the etiology of the disease in affected individuals, but also provides fertility guidance for this family.

## Data Availability

The datasets used and/or analyzed under the current study are available on reasonable and authentic request from the corresponding.
